# Adoption Dynamics of Organic Pesticides Among Cocoa Producers In Two Ecological Zones Of Ghana

**DOI:** 10.1002/gch2.202500045

**Published:** 2025-07-17

**Authors:** Michael Asigbaase, Simon Abugre, Mary Banowiiri, Josephine Akutteh

**Affiliations:** ^1^ Department of Forest Sciences University of Energy and Natural Resources Box 214 Sunyani Ghana

**Keywords:** adoption intensity, agroforestry, barriers, cocoa, perception, strategies

## Abstract

The use of organic pesticides to reduce insect and disease infestations and boost agricultural productivity can minimize the health and environmental costs of synthetic pesticides. However, adoption remains slow, and barriers and drivers influencing their uptake among cocoa farmers across different ecological zones are unclear. Grounded in the Diffusion of Innovations Theory, this study investigated perceptions, drivers, barriers, and strategies to enhance organic pesticide adoption among cocoa farmers in two ecological zones. A mixed‐methods approach is employed, collecting data from 450 farmers in eight cocoa‐growing communities through questionnaire‐led interviews. Data are analyzed using linear mixed‐effects regression, probit regression, ANOVA, Chi‐Square, and thematic analysis. Findings revealed that adopters have a 7%‐32% more favorable perception of the environmental and health benefits of organic pesticides, influencing their adoption. Farm characteristics, farming experience, incomes, land tenure, and ecological zone significantly influenced adoption. Non‐adopters cited barriers such as high transportation costs, offensive odors, and limited information access. Suggested strategies to enhance adoption included capacity building, financial incentives, improved product availability, institutional support, and awareness campaigns. These findings highlight the need for targeted interventions to address demographic and socio‐economic barriers and promote organic pesticide use. Future research should explore longitudinal impacts on productivity and soil health.

## Graphical abstract

The study explores the dynamics of organic pesticides among cocoa farmers, analyzing factors shaping farmers perception, adoption barriers and enablers, and factors driving the adoption of organic pesticides across different ecological zones. The findings highlight the diverse socio‐economic contexts within which cocoa operates and suggest targeted approaches are critically needed to address the specific needs and challenges of farmers to enhance the adoption of organic pesticides.

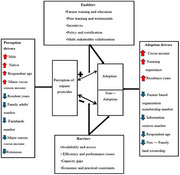

## 1 Introduction

Cocoa farming serves as a critical livelihood source for millions of smallholder farmers in tropical regions, contributing significantly to rural economies and global agricultural trade.^[^ ^1^, ^2^, ^3^, ^4^ ^]^ Cocoa farming, in particular, is a cornerstone of Ghana's agricultural sector, accounting for ≈20–30% of global cocoa production, and sustainable cocoa production remains a central focus due to its role in improving farmer incomes, conserving biodiversity, and mitigating climate change.^[^ ^5^, ^6^ ^]^ However, the sustainability of cocoa production in many countries, including Ghana, faces numerous challenges, such as pest and disease pressures that threaten yield and quality.^[^ ^4^, ^6^ ^]^ The conventional response to these challenges has been the widespread use of synthetic pesticides, which, while effective in the short term, present significant concerns regarding environmental degradation, human health risks, the loss of biodiversity, and long‐term soil productivity and economic viability of cocoa farming systems.^[^ ^7^, ^8^ ^]^ In response to these concerns, organic pesticides have been promoted as an eco‐friendly and sustainable alternative.^[^ ^9^, ^10^, ^11^ ^]^

International initiatives such as the Sustainable Development Goals (SDGs) and voluntary certification schemes like Rainforest Alliance and Fairtrade encourage the use of sustainable pest management practices, including organic pesticides.^[^ ^12^, ^13^, ^14^ ^]^ In Ghana, the government's policies on sustainable agriculture, coupled with programs promoting climate‐smart agriculture, provide a supportive framework for the adoption of organic pesticides.^[^ ^15^ ^]^ The adoption of organic pesticides is increasingly viewed as a critical pathway toward achieving sustainable cocoa production.^[^ ^11^, ^16^ ^]^ Organic pesticides, which are derived from natural substances, such as plant extracts, microorganisms, and minerals, have gained attention for their ability to manage pests while minimizing adverse environmental impacts.^[^ ^17^, ^18^, ^19^ ^]^ They align with the principles of sustainable agriculture, which emphasize maintaining ecological balance and reducing reliance on synthetic inputs. Organic pesticides also offer potential co‐benefits, including improved soil health, enhanced biodiversity, and reduced chemical residues in cocoa beans, which can increase market access to environmentally conscious consumers. However, despite these advantages, the adoption of organic pesticides among cocoa farmers remains limited, raising critical questions about the factors influencing their uptake, particularly in different ecological zones.^[^ ^7^ ^]^

Existing studies on cocoa production in Ghana have primarily focused on broader topics such as deforestation, carbon sequestration, and synthetic pesticide use.^[^ ^20^, ^21^, ^22^, ^23^ ^]^ While these studies provide valuable insights, they often overlook the dynamics of organic pesticide adoption. For instance, studies (e.g.)^[^ ^24^, ^25^, ^26^, ^27^, ^28^ ^]^ have highlighted farmers' awareness of organic farming practices, but they did not consider the specific perceptions, barriers, and enabling factors associated with organic pesticide adoption. Farmers’ perceptions of agricultural innovations play a critical role in shaping their adoption behavior.^[^ ^29^ ^]^ Perception encompasses farmers' understanding of the benefits, risks, and efficacy of organic pesticides in addressing pest and disease challenges. Perception shapes decision‐making, yet limited research has explored how cocoa farmers perceive the benefits, effectiveness, and cost implications of organic pesticides compared to synthetic alternatives.^[^ ^1^, ^30^ ^]^ Moreover, analyzing the drivers influencing these perceptions and identifying effective strategies to promote adoption can provide critical insights for policymakers, extension agents, and other stakeholders aiming to advance sustainable cocoa production practices.^[^ ^1^ ^]^

Furthermore, the socio‐economic and cultural drivers that influence the adoption of organic pesticides remain poorly understood, particularly in Ghana's diverse ecological zones, where farming practices and pest pressures differ.^[^ ^31^, ^32^ ^]^ Thus, a deeper understanding of the dynamics influencing the adoption of organic pesticides and the factors shaping farmers' decisions is urgently needed. That notwithstanding, farmers often face numerous challenges, including limited access to organic inputs, lack of technical knowledge, and inadequate market incentives.^[^ ^4^, ^6^, ^13^ ^]^ While these barriers are acknowledged in general agricultural contexts, specific studies on how they manifest in cocoa farming are scarce.^[^ ^4^ ^]^ The interplay between these barriers and ecological differences across cocoa‐growing regions further complicates the adoption landscape. Besides, strategies to enhance the adoption of organic pesticides have not been adequately explored.^[^ ^30^ ^]^

Research on farmers' adoption of sustainable agricultural practices has predominantly employed either qualitative or quantitative methods in isolation, often constraining the contextual depth or generalizability of findings.^[^ ^33^, ^34^ ^]^ Integrating both approaches through a mixed‐methods design offers a more comprehensive lens, particularly across diverse ecological settings where adoption dynamics vary significantly (Tashakkori & Teddlie, 2003). This methodological synergy is especially pertinent in agroforestry systems, where socio‐economic, ecological, and cultural dimensions converge in complex ways.^[^ ^33^, ^35^ ^]^

In addition to methodological integration, the use of advanced statistical techniques – such as linear mixed‐effects models (LMMs) – is essential for capturing the hierarchical structure of agroforestry data, which often exhibits spatial and temporal dependencies.^[^ ^36^, ^37^ ^]^ Despite their suitability, LMMs remain underutilized in agroforestry research within the global South. Applying this analytical framework to the study of organic pesticide adoption in cocoa systems presents a novel contribution, particularly in Ghana, where such approaches are rare. By combining qualitative insights with quantitative rigor and incorporating LMMs, this study provides a nuanced understanding of how individual decisions are shaped by broader environmental and socio‐economic contexts. This approach not only enhances the explanatory power of the findings but also ensures relevance to both policy and practice, addressing the complex realities faced by cocoa farmers in agroforestry landscapes.

Again, while policy frameworks such as the Cocoa and Forests Initiative emphasize sustainable cocoa production, actionable recommendations for promoting organic pesticide use are limited.^[^ ^38^, ^39^, ^40^ ^]^ Understanding how institutional support, training programs, and market incentives can drive adoption is crucial for scaling organic pesticide use among cocoa farmers. Therefore, the main objective of the study was to assess the perceptions, drivers, barriers, and potential strategies for enhancing the adoption of organic pesticides among cocoa farmers in two ecological zones. Specifically, the study sought to: (i) evaluate cocoa farmers' perception and the drivers of their perception of organic pesticides in different ecozones; (ii) determine the drivers of organic pesticide adoption and adoption intensity among cocoa farmers; (iii) identify and analyze barriers to the uptake of organic pesticides in cocoa farming in the two ecozones and explore strategies to enhance their adoption. The study is grounded in the Diffusion of Innovations theory, which posits that the adoption of new technologies is influenced by a combination of individual perceptions, social networks, and institutional support.^[^ ^41^, ^42^ ^]^ It is hypothesized that farmers with greater positive perception of the benefits of organic pesticides, access to training, and supportive institutional frameworks are more likely to adopt these practices. Conversely, challenges such as high costs, limited availability, and inadequate technical knowledge act as significant barriers to adoption. Wokibula et al.^[^ ^42^ ^]^ indicated that adopter and innovation characteristics influence adoption of innovations, but the role of ecological zones remains unclear.

Ghana's diverse ecological zones provide a unique opportunity to investigate the adoption dynamics of organic pesticides. These zones are characterized by distinct climatic conditions, soil properties, and vegetation types, which influence pest prevalence, disease dynamics, and the overall management practices of cocoa farms. Understanding how these ecological contexts affect farmers' perceptions, adoption drivers, and barriers to organic pesticide uptake is critical for designing targeted interventions to promote sustainable pest management practices. Empirical evidence on the adoption dynamics of organic pesticides is critical to inform policy and practice. Moreover, the study aligns with national and global efforts to achieve climate‐smart agriculture, enhance smallholder resilience, and reduce the environmental footprint of cocoa production.

## 2 Methodology

### 2.1 Study Site Description

**Figure 1:**
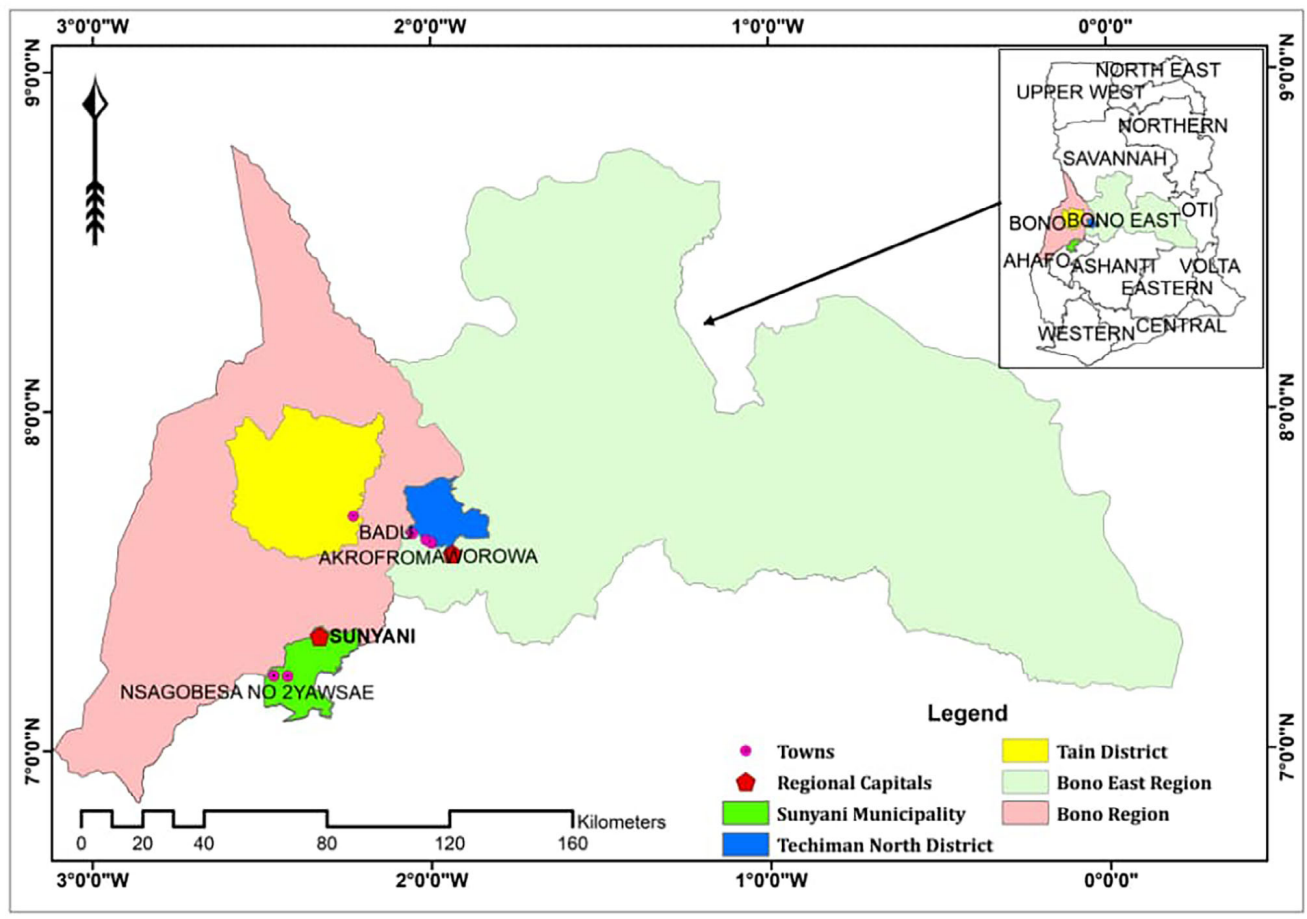
Study area map showing districts, major towns, and regional capitals.

The study was conducted in eight cocoa communities located in the Sunyani Municipality (Daadom, Nsagobesa, and Yawsae), Techiman North District (Aworowa, Atrensu, Akrofrum, and Gyebiri) and the Tain District (Badu). The Sunyani Municipality is situated between latitudes 7°20′N and 7°05′N and longitudes 2°30′W and 2°10′W in the Bono Region of Ghana (**Figure** **1**). The municipality covers an area of ≈828 km^2^. As of the latest census, the population stands at ≈147301, with a growth rate of ≈3.8%.^[^ ^43^ ^]^ The Techiman North District lies within the coordinates of latitude 7°40′N to 8°10′N and longitude 1°45′W to 2°10′W and it is located within the Bono East Region of Ghana (Figure 1). The district covers a total land area of ≈793 km^2^.^[^ ^43^ ^]^ The population of the Techiman North District is predominantly rural, with agriculture being the primary economic activity. The district experiences a moderate population growth rate, which impacts land use and agricultural practices.^[^ ^43^ ^]^ Covering an area of ≈4125 km^2^, the Tain District is one of the larger districts in the Bono Region. The district lies within latitudes 7°30′N and 8°45′N and longitudes 2°52′W and 0°28′E. The district shares an international border with Côte d'Ivoire to the northwest and is adjacent to the Bole District of the Northern Region to the northeast.

### 2.2 Climate, Vegetation, and Economic Activities

Tain and Techiman North Districts and Sunyani Municipality experience a tropical climate, characterized by two main seasons: the rainy season, which spans from April to October, and the dry season, from November to March. The average annual rainfall ranges from 1,200 to 1,500 mm in the Techiman North District and Sunyani Municipality, supporting diverse agricultural activities. The average annual rainfall ranges between 1000 and 1,400 mm in the Tain District. The monthly temperatures range from 23 to 33 degrees Celsius in all the study sites, with the coolest period occurring around August and the warmest temperatures observed in March and April. Relative humidity levels are between 75% and 80% during the rainy seasons and between 70% and 80% during the dry seasons. The vegetation in all the study sites was primarily made up of semi‐deciduous forest and savanna, providing a mix of forested areas and open lands suitable for cultivation.

Agriculture is the backbone of the local economy in the study sites. Cocoa and cashew are grown as cash tree crops. Other primary food crops cultivated include maize, cassava, yam, plantain, and vegetables. Additionally, the study areas are known for its vibrant poultry farming and livestock rearing activities.

### 2.3 Data Collection Approach

A multistage sampling approach was used to select participants for the study. Specifically, a random sampling technique was used to choose the ecozones, districts, or municipalities, and communities for this study. Initially, two cocoa‐producing ecozones in Ghana (Dry Semi‐Deciduous Zone – DSDZ, and Forest‐Savannah Transitional Zone, hereafter Transitional Zone – TZ) were randomly picked from a list of ecological zones. Subsequently, one municipality and one district within the Bono Region and one district in the Bono East Region were randomly selected. For the study, three cocoa‐growing communities (Daadom, Nsagobesa, and Yawsae) in Sunyani Municipality, four communities (Aworowa, Atrensu, Akrofrum, and Gyebiri) in Techiman district, and one community (Badu) in Tain district were randomly selected. Within each community, cocoa farmers were randomly chosen from a list provided by the local Ghana Cocoa Board offices, Purchasing Clerks, and Farmer‐based Organizations. Overall, 450 farmers were selected for the study: Daadom (62), Nsagobesa (48), Yawsae (44) Aworowa (46), Atrensu (51), Akrofrum (37), and Gyebiri (45) and Badu (117). Data collection was carried out through structured interviews using a questionnaire that covered demographic and socio‐economic information, perceptions of organic pesticides, levels of adoption, barriers to adoption, and strategies for improving organic pesticide use. The study adhered to local ethical guidelines established by the Ethics Committee of the Department of Forest Science, University of Energy and Natural Resources, as well as international standards outlined in the Helsinki Declaration of 1975 (revised in 2000). Participants were fully informed about the academic purpose of the research, and their consent was obtained prior to their involvement.

### 2.4 Data Processing and Statistical Analysis

The data gathered from the questionnaires and household interviews were coded and entered into a Microsoft Excel spreadsheet. After organizing the data in Excel, further analysis was performed using the Statistical Package for the Social Sciences (SPSS) version 25 for Windows. A perception index was estimated using Equation 1. Linear Mixed‐Effects Models were used to assess factors influencing the cocoa farmers’ perception and adoption intensity of organic pesticides. A probit analysis with a log likelihood‐link was used to determine the factors influencing the adoption of organic pesticides. Strategies to enhance the adoption of organic pesticides were analysed based on citation frequency. One‐way ANOVA and Chi‐Square were used to analyze the demographic and socio‐economic characteristics of cocoa farmers across ecological zones and rating scores of barriers to organic pesticides’ adoption. Model selection involved evaluating various combinations of independent variables using the Akaike Information Criterion (AIC) to determine the best‐fitting model. The model with the lowest AIC was deemed the best fit. Significance level was determined at α = 0.05. The qualitative data were systematically examined using thematic analysis, guided by the framework of Braun and Clarke,^[^ ^44^ ^]^ to identify and interpret key patterns related to farmers’ perceptions of strategies to enhance the adoption of organic pesticides.

(1) $$PI\left(\%\right)=\frac{\Sigma RS}{mn}\times 100$$

where PI = perception index, ∑RS = sum of rating scores of perceptions about organic pesticides, m = maximum rating score, and n = number of perception items.

## 3 Results and Discussion

### 3.1 Socio‐Economic Characteristics of Cocoa Farmers Across the eCological Zones

Cocoa farmers in both ecological zones were predominately males (2‐fold to 2.4‐fold higher), married (72%‐82%), Christians (82%‐90%) and natives (2.8‐fold in DSDZ and 1.3 in TZ) (**Table** **1**). Unmarried cocoa farmers in the TZ were 3‐fold more than that of DSDZ and TZ had relatively more migrants (≈2‐fold) than DSDZ. Relatively more (30%) cocoa farmers at TZ were landowners compared to those at DSDZ. However, land ownership at DSDZ generally was sharecropping, or family land. In general, these findings are consistent with broader gender dynamics and religious and rural cultural demographics of the ecozones.^[^ ^45^, ^46^ ^]^ Specifically, the predominance of men in cocoa farming is well‐documented in most cocoa producing countries in West Africa, where ownership and access to land and the physical demands of cocoa farming may favor men.^[^ ^45^, ^46^, ^47^, ^48^ ^]^ Additionally, marriage often provides social capital, support network, and labor that are essential in farming communities such as our study areas.^[^ ^45^ ^]^

**Table 1:** Socio‐economic characteristics of cocoa farmers across the ecological zones.

Category	Attribute	Ecozone			
		Dry Semi‐deciduous zone	Transitional zone	X^2^	P value
Gender	Female	70	71	0.659	0.416
	Male	141	169		
Marital status	Divorced	17	22	9.378	0.024
	Married	172	172		
	Single	9	28		
	Widower	13	18		
Origin	Migrant	56	105	15.024	< 0.001
	Native	155	133		
Religion	Christianity	188	194	11.867	0.007
	Islam	13	32		
	Other	7	7		
	Traditional	2	5		
Land tenure	Family land	44	62	25.288	< 0.001
	Owner	134	102		
	Rented	9	10		
	Share cropping	24	66		

On average, cocoa farmers at the DSD zone were older, resided longer in their communities, had more children, accessed credit more frequently, belonged to more FBOs, and received more extension support than those in the TZ (**Table** **2**). However, cocoa farmers at TZ were more educated, had older cocoa farms, and had more diverse sources of information than those at DSDZ. Again, TZ cocoa farmers had more spraying machines compared to those at the DSDZ. In relation to economic traits, TZ had significantly higher monthly cocoa income and overall annual income compared to DSDZ cocoa farmers. According to some studies (e.g.),^[^ ^45^, ^46^, ^49^ ^]^ FBOs often offer or facilitate access to inputs, credit, and information to its member, which are crucial for farmers in resource‐poor areas; this may explain the more significantly higher number of FBOs membership in the DSDZ. Moreover, the greater extension support observed in the DSDZ may engender FBO formation and participation as mechanisms to effectively disseminate information and provide support services. Studies have reported that extension services often promote FBOs as platforms for delivering agricultural innovations, services, and training.^[^ ^46^, ^49^ ^]^ The differences in income between cocoa farmers in the DSDZ and the TZ may be linked to various factors such as differences in productivity, market access, and community dynamics.^[^ ^49^ ^]^

**Table 2:** Mean (± SEM) socio‐economic traits of respondents across the ecozones.

Attribute	Ecozone (Mean ± SEM)		F‐value	P‐value
	Dry Semi‐deciduous zone	Transitional zone		
Residence years	25.35 ± 0.95	22.56 ± 0.93	4.333941	0.037931
Respondent age	49.03 ± 0.89	44.99 ± 0.63	14.26803	0.00018
Children number	2.97 ± 0.16	2.48 ± 0.11	6.977966	0.00854
Formal education years	4.92 ± 0.38	7.63 ± 0.39	24.49172	<0.0001
Spraying machines number	1.33 ± 0.05	1.65 ± 0.07	13.42851	0.000278
Cocoa farm age	17.67 ± 0.6	19.61 ± 0.67	4.503086	0.034387
Credit access frequency	1.8 ± 0.03	1.69 ± 0.03	7.457099	0.00657
Major season organic pesticide application (galoons)	2.17 ± 0.17	5.28 ± 0.91	8.865968	0.003085
Minor season organic pesticide application (galoons)	1.83 ± 0.15	2.97 ± 0.29	10.0658	0.001628
Organic pesticides application (yr^−1^)	3.25 ± 0.26	7.68 ± 1.1	13.69169	0.000242
Monthly cocoa income	542.54 ± 77.89	956.48 ± 116.92	7.983293	0.004943
Overall income (yr^−1^)	1235.1 ± 159.96	1699.77 ± 136.14	4.954967	0.026511
Information sources number	2.56 ± 0.09	2.89 ± 0.09	6.87675	0.009029
Extension support number (yr^−1^)	13.62 ± 0.94	5.7 ± 0.59	53.76319	<0.0001
FBO membership	1.23 ± 0.03	1.11 ± 0.02	8.239879	0.004295

### 3.2 Perceptions of Organic Pesticides Among Adopters and Non‐Adopters

In general, adopters of organic pesticides had a more favourable perception of organic pesticides compared to non‐adopters^[^ ^50^, ^51^ ^]^ (**Table** **3**).^[^ ^47^ ^]^ The higher ratings (7% – 32%) among adopters in this study reflect a strong belief in the health and environmental benefits of organic pesticides, which is in line with results from previous studies (e.g.).^[^ ^50^, ^51^ ^]^ This positive perception may possibly lead to the adoption of organic pesticides, as farmers who recognize their benefits are more likely to integrate them into their farming systems.^[^ ^46^, ^50^ ^]^ For instance, results from previous studies found that organic farmers place a high value on soil health and environmental stewardship, which significantly influences their farming practices.^[^ ^45^, ^46^, ^52^ ^]^ Besides, adopters may have had personal, or community values aligned with environmental conservation which motivates them to choose environmentally friendly practices, including the use of organic. Moreover, adopters may observe firsthand the benefits of organic pesticides in maintaining soil health, such as better soil structure, increased earthworm activity, and fewer pest outbreaks, reinforcing their positive perceptions.^[^ ^7^, ^45^, ^46^ ^]^

**Table 3:** Comparison of rating of adopters and non‐adopters’ perception of organic pesticides.

Perception	Adoption status (Mean ± SEM)		F‐value	P‐value
	Adopter	Non‐adopter		
Environmentally friendly	4.64 ± 0.03	4.14 ± 0.09	39.06985	<0.0001
Preserves soil organisms	4.42 ± 0.04	4.01 ± 0.10	20.85515	<0.0001
Improves cocoa seed quality	4.30 ± 0.04	3.85 ± 0.11	20.04961	<0.0001
Does not pollute water resources	4.20 ± 0.05	3.83 ± 0.11	11.51947	0.00075
Less costly	4.29 ± 0.05	3.26 ± 0.14	70.52462	<0.0001
More effective than synthetic pesticides	4.09 ± 0.05	3.82 ± 0.11	6.196069	0.01317
Offers longer protection against pest than synthetic pesticides	4.01 ± 0.05	3.55 ± 0.12	14.97303	0.000125
Always available	2.96 ± 0.07	3.18 ± 0.13	2.424265	0.120183
Smaller quantity required than synthetic pesticides	3.14 ± 0.06	3.13 ± 0.11	0.000338	0.98534
Less labor intensive	3.48 ± 0.07	3.28 ± 0.13	1.935391	0.164868
Less time consuming	3.40 ± 0.07	3.11 ± 0.12	4.138283	0.042515
No experience required	3.58 ± 0.06	3.66 ± 0.11	0.362618	0.547363
Health friendly	4.15 ± 0.05	3.74 ± 0.10	15.32981	0.000104
Reduces the incidence of weeds	3.35 ± 0.06	3.32 ± 0.11	0.046747	0.828922

Adopters of organic pesticides also perceived them as economically advantageous and efficient. They rated organic pesticides as less costly and time‐consuming, yet more effective and offering longer protection compared to synthetic alternatives. On the other hand, non‐adopters perceived organic pesticides as less cost‐effective or less efficient. The perception of adopters and non‐adopters was possibly linked to their demographic, socio‐economic, and farm characteristics.^[^ ^45^, ^51^ ^]^ However, both adopters and non‐adopters rated availability, ease of use, labor intensity, and required quantity of organic pesticides similarly, indicating common practical challenges. Specifically, organic pesticides may not be as readily available as synthetic ones, posing a challenge for both adopters and non‐adopters (Gunawan et al., 2022). Moreover, organic pesticides often require more precise application methods and timing, which can increase labor demands; both groups may perceive this as a drawback, particularly if labor is scarce or expensive.^[^ ^53^ ^]^

### 3.3 Factors Affecting the Perception of Organic Pesticides Among Cocoa Farmers

The linear mixed‐effects regression results (**Table** **4**) showed that farmers’ perception of organic pesticides was shaped by demographic, socio‐economic, and farm traits. Specifically, adopters, men, natives, and the age of cocoa farmers positively shaped their perception about organic pesticides, while years of residence, number of adult family members, and number of farmlands negatively influenced their perception. While cocoa yield during the major season negatively influenced the perception of organic pesticides, yield during the minor season positively influenced farmers’ perception about organic pesticides. Both credit access and extension support were negative predictors of perception about organic pesticides.

**Table 4:** Factors affecting cocoa farmers’ perception of organic pesticides.

Parameter	Mean [SEM]	Estimate/intercept [SEM]	df	P‐value
Intercept		41.38 ± 6.1	6.01E‐16	<0.0001
AdoptStatus = Adopter	44.33 ± 5.74	4.44 ± 1.22	231.2038	0.000348
AdoptStatus = Non‐adopter	39.88 ± 5.66			
Gender = Male	43.22 ± 5.67	2.23 ± 1.02	422.129	0.029747
Gender = Female	40.99 ± 5.71			
Origin = Native	44.23 ± 5.68	4.24 ± 1.06	356.1054	<0.0001
Origin = Migrant	39.98 ± 5.70			
Residence years	23.92 (na)	−0.10 ± 0.04	419.8697	0.018692
Respondent age	46.85 (na)	0.14 ± 0.05	422.2225	0.004344
Adult members number	2.93 (na)	−0.43 ± 0.24	417.3639	0.077412
Farm lands number	1.98 (na)	−1.73 ± 0.47	422.3835	0.000238
Major season cocoa harvest (bags)	10.98 (na)	−0.22 ± 0.08	422.894	0.004995
Minor season cocoa harvest (bags)	6.45 (na)	0.30 ± 0.11	422.6822	0.007674
Credit access frequency	1.38 (na)	−0.74 ± 0.44	422.6199	0.094092
Extension support frequency	9.21 (na)	−0.28 ± 0.05	303.5648	<0.0001

The fact that males and natives had higher perception scores regarding the benefits of organic pesticides compared to females and migrants is possibly because they had better access to agricultural resources, training, and extension services, which can enhance their understanding and appreciation of organic farming practices.^[^ ^42^, ^49^ ^]^ Furthermore, previous studies have shown that men and natives are usually more exposed to agricultural innovations and market dynamics due to their roles in farming operations and interactions with extension services.^[^ ^46^ ^]^ That notwithstanding, gender and nativity differences in perception may also reflect varying levels of risk tolerance.^[^ ^49^ ^]^ The fact that age positively influenced the perception of cocoa farmers may be related to concern for the environment and their health.

It was found that farmers with more land or larger farms had lower perception scores of organic pesticides, while major season cocoa yields negatively influenced, and minor season yields positively influenced perception scores. This observation may be as a result of several factors. For example, larger farms often face greater logistical challenges and higher costs in adopting organic practices, which can be labour‐intensive and require more inputs per unit area.^[^ ^54^ ^]^ This can deter farmers with larger farmlands from adopting organic pesticides. Furthermore, farmers with larger holdings and higher yields may prioritize high‐output farming systems, which usually rely on synthetic inputs to maximize productivity, making them less inclined to adopt organic practices.^[^ ^47^, ^54^, ^55^, ^56^ ^]^ The positive correlation between minor season yields and perception of organic pesticides may be due to lower pest pressure in this season. The positive influence of age on the perception of organic pesticides suggests that older farmers may have a greater awareness of the long‐term benefits.

### 3.4 Factors Influencing the Adoption of Organic Pesticides Among Cocoa Farmers

The probit regression results showed native cocoa farmers and those in the DSDZ were 56% and 184%, respectively, less likely to adopt organic pesticides (**Table** **5**). This possibly reflects the variation in extension support, ownership of agricultural tools, access to markets, social network dynamics, and diversity of information sources across the ecozones (Table 2 ^[^ ^42^, ^46^ ^]^). Furthermore, older farmers, farmers of older cocoa farms, and those with a larger number of adult members were less likely to adopt organic pesticides (Table 5; Djokoto et al., 2016;).^[^ ^42^ ^]^ These findings support the notion that age is negative predictor of adoption of innovations (e.g.;^[^ ^57^ ^]^ Djokoto et al., 2016) but diverges form reports of previous studies within the agricultural space, where older farmers were more inclined to adopt sustainable farming practices, including organic pesticides and integrated pest management strategies (e.g.^[^ ^54^, ^58^ ^]^). However, farmers who had lived longer in their respective communities and experienced cocoa farmers were, 3% and 1.6% respectively, more likely to adopt organic pesticides highlighting the role of experiences in understanding the benefits of organic practices such as soil health and sustainability.^[^ ^46^ ^]^ Our results are in line with Ullah et al.^[^ ^59^ ^]^ and Sapbamrer & Thammachai,^[^ ^53^ ^]^ who argued that experienced farmers were more likely to adopt organic farming due to extensive knowledge, concern about the environment or better access to organic markets and premium prices. Additionally, higher off‐farm and cocoa incomes were associated with a greater likelihood of adopting organic pesticides, which possibly reflects factors such as financial stability, risk mitigation, or access to resources.^[^ ^46^, ^52^ ^]^ For example, farmers with off‐farm income are better positioned to absorb potential yield losses, as they are not solely reliant on cocoa farming for their livelihood.^[^ ^46^, ^52^ ^]^ Again, income diversification allows farmers to experiment with new practices without significant adverse impacts on their household's financial security.^[^ ^47^ ^]^ That notwithstanding, the minimal effect of higher cocoa income on adoption implies that income from cocoa alone may not be a strong driver for adopting organic pesticides. On the contrary, a higher number of information sources and membership in FBOs significantly reduced the likelihood of adopting organic pesticides (Table 5). Furthermore, a higher perception index, reflecting a positive attitude toward organic pesticides, was associated with increased adoption. Different land tenure systems showed varying influence on the adoption of organic pesticides; rented land decreased the likelihood of adoption with a greater magnitude than sharecropping or family land.^[^ ^59^ ^]^

**Table 5:** Factors influencing the adoption of organic pesticides among cocoa farmers.

Parameter	Estimate/intercept [SEM]	Wald Chi‐Square	P‐value
(Intercept)	4.23 ± 1.17	13.09503	0.000296
EcoZone = Dry Semi‐deciduous	−1.84 ± 0.28	44.0704	<0.0001
EcoZone = Transitional			
Origin = Native	−0.56 ± 0.21	7.209942	0.00725
Origin = Migrant			
Residence years	0.03 ± 0.01	7.293079	0.006922
Respondent age	−0.07 ± 0.02	21.42449	<0.0001
Adult members number	−0.1 ± 0.05	3.799332	0.051273
Cocoa farming years	0.07 ± 0.02	17.1563	<0.0001
Cocoa farm age	−0.02 ± 0.01	3.790388	0.051548
Credit access frequency	0.19 ± 0.11	2.953831	0.085674
Monthly cocoa income	1.58 × 10^−4^ ± 8.31 × 10^−5^	3.602249	0.057701
Monthly off‐farm income	1.46 × 10^−4^ ± 5.20 × 10^−5^	7.84593	0.005094
Information sources number	−0.21 ± 0.08	7.702711	0.005514
FBO membership	−1.65 ± 0.56	8.70565	0.003172
Perception index	0.03 ± 0.01	10.70243	0.00107
Land tenure = Owner	−0.23 ± 0.26	0.779455	0.377308
Land tenure = Rented	−1.09 ± 0.54	4.131459	0.042093
Land tenure = Share cropping	−0.49 ± 0.28	3.009615	0.082772
Land tenure = Family land			
Religion = Christianity	−1.44 ± 0.66	4.72235	0.029773
Religion = Islam	−1.33 ± 0.7	3.671906	0.055337
Religion = Other	−1.02 ± 0.8	1.63216	0.201405
Religion = Traditional			
Farmlands number	0.05 ± 0.09	0.311525	0.576746
Extension support frequency	0.01 ± 0.01	1.511852	0.218857

### 3.5 Factors Affecting the Intensity of Organic Pesticides Adoption Among Cocoa Farmers

The intensity of adoption of organic pesticides among the cocoa farmers was influenced by land tenure, socio‐economic, farm, and demographic traits (**Table** **6**). Specifically, farmers owning their land is a major significant predictor of adoption intensity of organic pesticides, possibly due to the security of land tenure.^[^ ^11^ ^]^ This implies that policies aimed at increasing land tenure security, particularly for sharecroppers and tenants, may encourage the adoption of sustainable practices.^[^ ^3^ ^]^ Additionally, providing financial and technical support tailored to the needs of non‐landowning farmers may help bridge the adoption gap.^[^ ^46^ ^]^ Larger household, higher education level, higher number of information sources, farmers of older cocoa farms, and longer residence positively influenced the adoption intensity of organic pesticides.^[^ ^47^ ^]^ This is because educated farmers were more likely to be aware of the benefits and correct use of organic pesticides, access information, and understand the health and environmental implications of chemical pesticides.^[^ ^46^ ^]^ Educated farmers are more likely to seek out and utilize new agricultural technologies and practices.^[^ ^46^, ^47^ ^]^ Additionally, larger households may provide more labour, facilitating the intensive use of labour‐intensive practices like organic pesticides.

**Table 6:** Factors affecting the intensity of adoption of organic pesticides.

Parameter	Mean [SEM]	Estimate/ intercept [SEM]	df	P‐value
Intercept		−9.91 ± 3.65	266.91	0.00702
Land tenure = Owner	6.98 ± 0.95	4.15 ± 1.55	420.67	0.007578
Land tenure = Rented	4.95 ± 2.91	2.12 ± 3.11	421.48	0.495989
Land tenure = Share cropping	5.19 ± 1.42	2.36 ± 1.82	382.03	0.195263
Land tenure = Family land	2.83 ± 1.31			
Household size	5.37 (na)	0.45 ± 0.21	411.34	0.03404
Residence years	23.95 (na)	0.16 ± 0.06	421.99	0.004554
Children number	2.71 (na)	−0.6 ± 0.37	420.72	0.104478
Formal education years	6.32 (na)	0.47 ± 0.11	409.14	<0.0001
Cocoa farming years	17.74 (na)	−0.42 ± 0.1	416.76	<0.0001
Cocoa farm age	18.87 (na)	0.34 ± 0.09	374.12	0.000151
Access to credit frequency	1.75 (na)	2.29 ± 1.42	420.73	0.107465
Information sources number	2.74 (na)	1.42 ± 0.46	393.59	0.00225
Extension support frequency	6.44 (na)	−0.27 ± 0.11	286.85	0.017536

*Note*: na means not applicable.

Surprisingly, the study found a negative influence of extension support on the intensity of organic pesticide adoption which contrasts previous findings,^[^ ^53^, ^54^, ^60^, ^61^, ^62^, ^63^, ^64^, ^65^ ^]^ possibly because extension services in the area may not prioritize or adequately promote organic practices. Alternatively, it might reflect a lack of trust in extension agents or perceived bias toward conventional methods. The positive influence of the number of sources of information on organic pesticide adoption suggests that access to diverse information channels enhances farmers’ knowledge and confidence in adopting organic practices.^[^ ^53^ ^]^

### 3.6 Barriers to the Adoption of Organic Pesticides Among Cocoa Farmers Across the Ecozones

**Table** **7** presents a detailed analysis of the perceived barriers to the adoption of organic pesticides among cocoa farmers. High transportation costs emerged as a significant barrier, particularly for non‐adopters who rated it substantially higher than adopters; this finding is in line with existing literature (Oyedele et al., 2018; Khoy et al., 2017; Pandey and Singh, 2012). Cocoa farms are generally located in remote areas with limited infrastructure, leading to increased costs for transporting organic pesticides.^[^ ^46^ ^]^ This implies policies and strategies aimed at lowering transportation costs among cocoa farmers, such as improved road network and cooperative bulk buying may potentially enhance the adoption intensity of eco‐friendly agricultural practices such as organic pesticides.^[^ ^66^ ^]^ While not often highlighted in agricultural technology adoption studies, sensory perceptions like odour potentially affect farmers’ willingness to work with certain products.^[^ ^46^ ^]^ The 23% higher concern among non‐adopters about organic pesticides having an offensive odour supports this notion. This perceived sensory concern can deter farmers from using these products, especially if the odour is strong enough to affect the working environment. The offensive odour of organic pesticides may be due to the natural ingredients used, which can be more pungent than synthetic chemicals. Besides, non‐adopters rated organic pesticides higher as having a slow effect (18%) and being less effective (20%) compared to adopters, possibly indicating a major concern regarding the perceived effectiveness of these products.

**Table 7:** Comparison of the rating of barriers to the adoption of organic pesticides among cocoa farmers in different ecological zones.

Perceived constrain	Adoption status [Mean ± SEM]		F‐value	P‐value
	Adopter	Non‐adopter		
Slow effect on pest and diseases	2.54 ± 0.07	3.09 ± 0.13	14.50422	0.00016
High transportation cost	2.98 ± 0.07	3.74 ± 0.13	30.46125	<0.0001
Offensive odour	2.53 ± 0.07	3.11 ± 0.14	15.5068	<0.0001
Unavailability	4.02 ± 0.06	3.88 ± 0.12	1.483228	0.223916
Difficult to access	3.97 ± 0.05	3.87 ± 0.12	0.689964	0.406622
Labor intensive	3.12 ± 0.07	3.04 ± 0.13	0.303245	0.582132
Large quantities required	3.38 ± 0.06	3.02 ± 0.13	7.211463	0.007515
Less effective	2.32 ± 0.07	2.78 ± 0.12	10.50595	0.00128
Risky to use	2.32 ± 0.07	2.78 ± 0.14	10.54402	0.001254
Limited agrochemical shops	4.03 ± 0.06	3.88 ± 0.12	1.495988	0.221938
Limited access to information	4.09 ± 0.05	3.67 ± 0.12	14.10223	0.000196
Limited training	4.27 ± 0.05	3.65 ± 0.12	29.83993	<0.0001

Adopters of organic pesticides rated access to information and limited training as more significant barriers than non‐adopters, possibly because dissemination of information about organic pesticides is often inadequate, with farmers relying on informal sources or anecdotal evidence, which may not be accurate or comprehensive.^[^ ^3^ ^]^ Access to information and training is a common barrier to the adoption of agricultural practices in many cocoa‐producing countries, including Ghana.^[^ ^47^ ^]^ Furthermore, adopters expressed more concern about the need for large quantities of organic pesticides per unit area, with a 10% higher concern compared to non‐adopters. This perceived barrier possibly reflects the practical challenges of sourcing and applying sufficient quantities of organic pesticides, which may be more voluminous or less concentrated than synthetic alternatives (Berhance et al., 2015; IFOAM, 2008). The logistical and financial burden of handling larger volumes can be a deterrent, especially for smallholder farmers.^[^ ^51^ ^]^

Both adopters and non‐adopters rated other constraints such as unavailability, difficulty accessing products, labour‐intensive application processes, riskiness of use, and limited availability in agrochemical shops, similarly.^[^ ^45^, ^46^ ^]^ This uniformity suggests that these perceived barriers are possibly common concerns irrespective of adoption status. Such barriers highlight the systemic issues in the agricultural input supply chain and the need for broader structural improvements to support sustainable farming practice.^[^ ^38^, ^49^ ^]^

### 3.7 Strategies to Enhance the Adoption of Organic Pesticides Among Cocoa Farmers in the Two Ecological Zones

Farmer training and education, access and availability, and peer learning and testimonials were strongly emphasized by cocoa farmers in both the Forest and Transitional zones (**Table** **8**).^[^ ^67^ ^]^ This possibly reflects a broader demand for capacity building in understanding and implementing organic practices.^[^ ^68^ ^]^ The centrality of training in both zones corroborates previous reports from Indonesia,^[^ ^16^, ^69^ ^]^ Colombia,^[^ ^70^ ^]^ and India.^[^ ^68^ ^]^ Moreover, Kassie et al.^[^ ^67^ ^]^ identified farmer education as a fundamental driver of technology adoption across diverse agricultural settings. Demonstration farms provide practical evidence of the effectiveness of organic pesticides, helping to build trust among farmers.^[^ ^47^, ^67^ ^]^ For example, in Nigeria, demonstration farms have been used to educate farmers about sustainable farming techniques and to demonstrate the practical benefits.^[^ ^71^, ^72^ ^]^ Additionally, access to organic inputs is reportedly a critical factor influencing their adoption in many agrarian economies.^[^ ^73^ ^]^ Therefore, the shared emphasis on training, access, and peer learning indicates that these three strategies form the core pillars of an effective organic pesticide adoption framework within the ecozones and suggests a strong potential for scalability of intervention models across Ghana and possibly other cocoa‐producing countries with similar dynamics.^[^ ^74^, ^75^ ^]^ This suggests that national or regional interventions can leverage these commonalities for broader implementation, even while adapting peripheral strategies to local contexts. However, assessing the causal impact of these commonly emphasized strategies on adoption rates and examining how combinations of training, access, and peer support can be bundled for maximum effect is urgently needed.

**Table 8:** Strategies to enhance the adoption of organic pesticides among cocoa farmers across the ecological zones.

Strategy Category	Key Actions Included	Illustrative Quote	Frequency	
			Forest Zone	Transitional Zone
1. Farmer Training and Education	Regular workshops, field demonstrations, extension visits, cooperative meetings	“When the extension officer showed me step‐by‐step during the workshop, I finally understood how to mix and spray properly.”— Female, Native	123	122
2. Access and Availability	Ensuring agro‐shop stocking; timely distribution via COCOBOD or local outlets	“When it is easy to find organic pesticides to buy, it will help a lot.”— Male, Migrant	70	42
3. Peer Learning and Testimonials	Past‐user sharing, lead‐farmer demo plots, published success stories	“After seeing organic pesticides work well in my neighbour's farm, I was convinced to give it a try.” — Male, Native	42	42
4. Local Production and Resources	Planting neem/other organic pesticide species; village‐level extract presses	“If they show us how to extract the neem and use and help us with the tools we need, I will try it” — Female, Native	50	18
5. Subsidies, Incentives and Credit	Subsidized “starter kits,” premium prices for organic cocoa, microcredit links	“Getting the bottle and sprayer on credit or for free will make all the difference—I could afford to experiment.” — Male, Migrant	20	36
6. Awareness and Advertising	Radio/announcements, church/school campaigns, social‐media ads	“When they make announcement on the radio about organic pesticides, more farmers will hear about it and try it.” — Female, Native	15	32
7. Multi‐stakeholder Collaboration	Partnerships among COCOBOD, NGOs, researchers, dealers	“Support from NGOs and COCOBOD means we can have correct information and quality inputs.” — Male, Native	25	13
8. Policy and Certification	Simplified organic labels, government support programs, certification schemes	“Mass spraying like the one they do for synthetic pesticides will be very helpful and many people will try it.” — Female, Migrant	16	15

Finally, the support for supportive policies and certification systems in both zones implies that institutional frameworks—such as mass spraying programs for organics or simplified certification for smallholders—can drive broader uptake. Policy support is essential for creating a favourable regulatory environment that encourages the adoption of organic pesticides, such as through subsidies, certification programs, and other incentives.^[^ ^67^ ^]^ Studies such as Setboonsarng^[^ ^76^ ^]^ and Willer and Lernoud^[^ ^77^ ^]^ have shown that streamlined organic certification processes significantly improve smallholder participation in organic markets. Certification provides access to niche markets that are willing to pay premium prices for certified organic products.^[^ ^49^ ^]^ For example, in Ghana and Cote d'Ivoire, certification programs have been key in accessing premium markets for organic cocoa.^[^ ^49^ ^]^

In addition to strategies commonly emphasized across both ecological zones, cocoa farmers in the Forest Zone demonstrated distinct support for local production and resources (50 mentions in Forest vs 18 in Transitional) and multi‐stakeholder collaboration (25 vs 13). Leeuwis and Aarts^[^ ^78^ ^]^ stressed that innovations in agriculture are more likely to succeed when embedded in co‐learning and collaborative networks. Thus, the emphasis to multi‐stakeholder collaboration in the Forest Zone indicates that successful interventions should build on integrated efforts involving research institutions (e.g., CRIG), NGOs, and public agencies (e.g., COCOBOD).

The Transitional Zone's higher emphasis on awareness and advertising, subsidies, and credit access reflects broader findings in smallholder agricultural literature. Studies such as Marenya and Barrett^[^ ^79^ ^]^ have shown that liquidity constraints often inhibit technology adoption among resource‐poor farmers. Similarly, Dormon et al.,^[^ ^80^ ^]^ in their study of cocoa farmers in Ghana, observed that upfront costs of inputs frequently discourage experimentation with unfamiliar technologies like organic products. Our findings imply that scaling organic pesticide adoption in the Transitional Zone may be more successful if paired with targeted financial incentives and robust public awareness efforts. The strong preference for subsidies and credit‐based models indicates that without reducing the financial risks, many farmers may remain hesitant to transition from conventional pesticides. This aligns with observations by Kuhlman et al.,^[^ ^81^ ^]^ who found that economic incentives can effectively promote environmentally sustainable behavior among farmers, particularly in contexts where cash flow is limited. Moreover, the Transitional Zone's emphasis on awareness and advertising highlights the role of information asymmetry in technology diffusion. Advertising serves to raise awareness and motivates farmers by showcasing the benefits of adopting organic pesticides, potentially overcoming resistance due to unfamiliarity.^[^ ^3^ ^]^ For example, advertising through local media and community meetings has been identified as a key strategy for disseminating information about new agricultural technologies and products.^[^ ^82^, ^83^ ^]^ Well‐targeted information campaigns can catalyse behavior change in integrated pest management adoption.^[^ ^84^ ^]^ Additionally, access to organic inputs is reportedly a critical factor influencing their adoption in many agrarian economies, including Ghana.^[^ ^73^, ^85^ ^]^ In line with Rogers’ (2003) diffusion of innovation theory, visibility and trialability of innovations are key drivers of adoption.

## 4 Conclusion 

By integrating qualitative and quantitative data within a linear mixed‐effects modeling framework, this study introduced a novel methodological approach that captured the hierarchical and context‐dependent drivers of organic pesticide adoption in cocoa agroforestry. The socio‐economic, demographic, and farm characteristics differed between cocoa farmers across ecological zones. DSDZ cocoa farmers of organic pesticides were older with larger families and more institutional support, while TZ cocoa farmers and non‐adopters were more educated and had more agricultural resources, which translated into higher cocoa yields and incomes. These differences influenced their perception and adoption of organic pesticides, with the key predictors being gender, nativity, credit support, extension support, land tenure, and number of sources of information. The identified key predictors offer empirically grounded insights with relevance for cocoa‐growing regions across West Africa. With regards to barriers to adoption of organic pesticides, practical implementation issues such as its effectiveness, costs, and odour were the major constraints among non‐adopters. However, the major constraints among adopters were related to capacity building and access to information. Consequently, institutional support such as research and development, technical support, incentives, regular training, and policy support was perceived as being critical to promoting organic pesticides. These findings highlight the diverse socio‐economic contexts within which cocoa operates and suggest targeted approaches are critically needed to address the specific needs and challenges of farmers to enhance the adoption of organic pesticides.

## Conflict of Interest

The authors declare no conflict of interest.

## Author Contributions

M.A. did conceptualization and methodology. M.A. did formal analysis and investigation. M.A. did Writing – original draft preparation. M.A., M.B., and J.A. did Writing – review and editing. M.A., M.B., S.A., and J.A. did resources. M.B. and M.A. did supervision and project administration. M.A. and S.A. did curation and visualization.

## Ethical Considerations

The research adhered to the guidelines of the Helsinki Declaration of 1975 (revised in 2000) and the Ethics Committee of the Department of Forest Science, University of Energy and Natural Resources. The informed of all participants was obtained before they were engaged. All participants were informed of the intention to publish the results of the study and they contented prior to their participation.

## Data Availability

The data that support the findings of this study are available from the corresponding author upon reasonable request.
